# MicroRNA-Mediated Post-Transcriptional Regulation of Enzymes Involved in Herbicide Resistance in *Echinochloa oryzicola* (Vasinger) Vasinger

**DOI:** 10.3390/plants14050719

**Published:** 2025-02-26

**Authors:** Carlo Maria Cusaro, Enrica Capelli, Anna Maria Picco, Marta Guarise, Enrico Gozio, Pietro Zarpellon, Maura Brusoni

**Affiliations:** 1 Department of Earth and Environmental Sciences, University of Pavia, Via S. Epifanio 14, 27100 Pavia, Italy; carlomaria.cusaro01@universitadipavia.it (C.M.C.); enrica.capelli@unipv.it (E.C.); annpic04@unipv.it (A.M.P.); 2 Agricola 2000 S.c.p.A., Via Trieste, 9, 20067 Tribiano, Italy; m.guarise@agricola2000.com (M.G.); e.gozio@agricola2000.com (E.G.); p.zarpellon@agricola2000.com (P.Z.)

**Keywords:** late-watergrass, profoxydim resistance, microRNA, CYP450, GST, eIF4B, CYP81A

## Abstract

Herbicide resistance is an emerging phytosanitary threat, causing serious yield and economic losses. Although this phenomenon has been widely studied, only recently has the role of epigenetic factors in its occurrence been considered. In the present study, we analyzed the microRNA-mediated regulation in *Echinochloa oryzicola* (Vasinger) Vasinger (late-watergrass) of the expression of cytochromes P450, glutathione S-transferase (GST), and eIF4B, all of which are enzymes involved in profoxydim (AURA^®^) detoxification. Before and after profoxydim application, the expression profiles of microRNAs (miRNAs) were selected for their ability to target the genes considered, and their targets were assessed by means of RT-qPCR. Susceptible and resistant biotypes showed different responses to this herbicide. After profoxydim application, in resistant biotypes, *osa-miR2099-5p*, *ath-miR396b*, *osa-miR395f*, *osa-miR396a-5p*, *osa-miR166a-5p*, *osa-miR166d-5p*, *gra-miR8759*, and *gma-miR396f* were not triggered, allowing the expression of CYP81A, *GSTF1*, and *eIF4B* genes and the herbicide’s detoxification. Meanwhile, the transcription of *ata-miR166c-5p*, *ath-miR847*, *osa-miR5538*, and *gra-miR7487c* was triggered, down-regulating *CYP71AK2*, *CYP72A254*, *CYP72A122*, and *EcGST* expression. In susceptible biotypes, the herbicide stimulated *ata-miR166c-5p*, *ath-miR847*, *osa-miR5538*, *gra-miR7487c*, *osa-miR166a-5p*, and *gra-miR8759*, down-regulating their respective target genes (*CYP72A122*, *CYP71AK2*, *EcGST*, *CYP72A254*, *CYP81A12*, and *eIF4B*). A better understanding of the role of miRNA-mediated epigenetic regulation in herbicide resistance will be useful in planning more targeted and sustainable methods for controlling this phytosanitary threat.

## 1. Introduction

Italy is the largest rice (*Oryza sativa* L.) producer in Europe. In 2020, the cultivated area was approximately 500 thousand hectares, providing a yield of 1.5 million tons, representing 53% of Europe’s total rice production [1,2]. Therefore, rice production is one of the main Italian agri-food sectors. The most important agricultural districts suited to rice cultivation are Piedmont and Lombardy (Po Valley, northern Italy). Here, herbicide resistance (HeR) has become a serious problem, causing yield losses [3,4].

According to the Herbicide Resistance Action Committee (HRAC), resistance to herbicides is an example of the adaptive evolution of weeds in response to human selection pressures, a consequence of the ability of plants to survive the application of chemicals that normally would kill them [5].

Over the years, the increasing prevalence of repeated field applications of an increasingly narrow range of herbicides, as a consequence of the withdrawal of many plant protection products (PPPs) from the EU market due to strict regulations (Reg. EC/1107/2009), and the lack of herbicides with new modes of action (MoAs), has led to a continuous rise in and spread of resistance [6,7,8].

Referring to the International Herbicide-Resistant Weed Database, there are currently 523 weed species (272 dicots and 251 monocots) reported as resistant to the most commonly used herbicides. Weeds have evolved resistance to 21 out of the 31 known MoAs and to 167 different herbicides. Herbicide-resistant weeds have been reported for 99 crops in 72 countries [9].

The development and occurrence of herbicide resistance could be due to missense DNA mutations, which cause target site resistance (TSR), or to the physiological degradation processes of the herbicide, which cause non-target site resistance (NTSR). A series of proteins, such as cytochromes P450 (CYP450) and glutathione S-transferase (GST), are involved in the physiological degradation process of herbicides, inactivating and isolating them [10,11,12].

The family of cytochromes P450 includes multiple genes that facilitate the denaturation of a wide range of chemicals, encoding heme-dependent enzymes that catalyze oxygen and NADPH-dependent monooxygenation reactions. The substrate specificity of P450 enzymes is high. However, each CYP450 tends to metabolize only a limited number of herbicides, particularly phenylureas. CYP450 belonging to the CYP81A subfamily are an exception, as they are involved in the metabolism of numerous herbicides in *Poaceae* crops [11,13]. For instance, *CYP81A6* is involved in tolerance to bentazone and to several sulfonylurea herbicides in rice (*O. sativa*) [14]; *CYP81A9* metabolizes sulfonylurea, benzothiadiazinone, and triketone in corn (*Zea mays* L.) [15,16]; and *CYP81A63* is involved in the detoxification of three chemical groups of acetyl-CoA carboxylase (ACCase) inhibitors in barley (*Hordeum vulgare* L. ‘Golden Promise’), namely phenylpyrazoline (pinoxaden), cyclohexanedione (tralkoxydim), and aryloxyphenoxypropionate (diclofop-methyl) [17]. Other members of the CYP81A subfamily were reported to be involved in herbicide detoxification in *Echinochloa*, particularly in *Echinochloa phyllopogon* (Stapf) Vasc. Specifically, the overexpression of *CYP81A12* and *CYP81A21* was responsible for cross-resistance to aceto-lactate-synthetase (ALS) and to acetyl CoA carboxylase (ACCase) inhibitors [18]. *CYP81A24* was found to metabolize pinoxaden and tralkoxydim (ACCase inhibitors) in *E. phyllopogon*, indicating that these enzymes are crucial in the occurrence of herbicide resistance in this weed [19].

Glutathione S-transferases (GST) also act in the herbicide detoxification process, following the oxidation of the substrate by cytochromes P450, by catalyzing the conjugation of the various oxidated substrates with glutathione to form a polar S-glutathionylated product (R-SG), thus reducing the reactiveness of the chemical to be detoxified [11]. The involvement of GST enzymes in the detoxification of ACCase inhibitor herbicides was recently reported in Chinese *Echinochloa glabrescens* Munro ex Hook (*Echinochloa crus-galli* (L.) P. Beauv. var. *formosensis*) [20] and in Chinese *E. phyllopogon* against metamifop (chemical group: aryloxyphenoxypropionate) [21].

The *eIF4B* gene encodes for an RNA-binding protein involved in the regulation of the initiation stage of protein synthesis [22]. Its overexpression is related to the occurrence of stresses in plants and culminates in a higher content of detoxifying enzymes, such as CYP450 and GST, leading to herbicide detoxification [23]. Thus, the expression of the enzymes involved in herbicide metabolic degradation is triggered by the application of chemical control, which stimulates an adaptive response in the plant.

Although herbicide detoxification by CYP450 and GST has been assessed in *Echinochloa* [14,15,16,17,18,19,20,21], only recently has the scientific community started to consider the role of epigenetics in the occurrence of herbicide resistance [24].

Small non-coding RNAs (sncRNAs), as epigenetic modulators [25,26], act at the post-transcriptional level by pairing with messenger RNA (mRNA) transcripts, inhibiting or down-regulating gene expression depending on the perfect or near-perfect complementarity [27,28,29]. It is well known that microRNAs (miRNAs) are conserved across species and kingdoms [30]. For example, plants and animals share miRNAs of the miR854 family [31]. The transcription of miRNAs is triggered by a variety of environmental signals, including ecological stressors, like heat [32], cold [33], drought [34], and salinity [35], producing a stress response that allows the organism to tolerate adverse conditions. Recently, the transcription of some miRNAs enabled the down-regulation of the expression of CYP450 and GST genes after the application of bispyribac-Na was observed in Italian-resistant biotypes of *E. crus-galli* [36].

The species of the genus *Echinochloa* (L.) P. Beauv. are widely distributed in Italian paddy fields, causing serious infestation problems [4]. These weeds are particularly noxious due to their evolution of resistance against the most commonly used herbicides [3]. *Echinochloa oryzicola* (Vasinger) Vasinger (late-watergrass) is one of the most problematic. It is an annual allo-tetraploid weed (2n = 4X = 36) native to Asia that has now spread all over the world, from tropical to temperate regions [37]. While adapting to a wide range of natural environments, *E. oryzicola* prefers flooded rice fields. Cases of cross-resistance have been reported for *E. oryzicola* [38].

One of the herbicides mainly used to control *Echinochloa* species is AURA^®^ (profoxydim—chemical group: cyclohexanedione—HRAC group 1), an inhibitor of the production of the ACCase enzyme. Due to its harmful effects on aquatic organisms and its persistence in the environment, it was banned in the EU in November 2021, pursuant to Regulation (EC) No. 1107/2009 of the European Commission [6]. However, due to the phytosanitary emergency regarding the need to control *E. crus-galli*, *Echinochloa oryzoides* (Ard.) Fritsch, *Echinochloa colona* (L.) Link, and *Panicum dichotomiflorum* Michx., the use of AURA^®^ (profoxydim) in Italy was allowed up until 2024, pursuant to art. 53 of Regulation (EC) No. 1107/2009 [6,39,40,41].

In our research, we wanted to assess the role of miRNAs in the regulation of the expression of genes involved in profoxydim resistance in *E. oryzicola*. The expression of genes encoding for cytochromes P450 monooxygenase and glutathione S-transferase and the transcription of miRNAs targeting their messenger RNAs was analyzed in Italian *E. oryzicola* biotypes resistant to profoxydim. We recorded the expression of nine CYP450 (*CYP81A12*, *CYP81A21*, *CYP81A22*, *CYP81A24*, *CYP81A63*, *CYP81A6*, *CYP71AK2*, *CYP72A122*, and *CYP72A254*), two GST (*EcGST* and *GSTF1*), and the eukaryotic translation initiation factor 4B (*eIF4B*) in *E. oryzicola* specimens collected from paddies in the Lombardy region (Po Valley, northern Italy). The expression profiles of CYP450 monooxygenase, GSTs, and eIF4B were analyzed in relation to some miRNAs able to target their transcript sequences (mRNAs). The miRNAs selected for analysis in our present research were predicted in silico by means of the psRNATarget: A Plant Small RNA Target Analysis web server [42,43,44] and selected on the basis of their high sequence complementarity with the transcripts of CYP450, GST, and eIF4B genes. The expression profiles of the miRNAs considered and their target genes were evaluated in herbicide-sensitive and -resistant late-watergrass biotypes by means of relative real-time quantitative PCR (RT-qPCR) before and after herbicide administration. Our findings highlighted a miRNA-mediated regulation of *E. oryzicola*’s response to profoxydim treatment.

## 2. Results

The profoxydim susceptibility/resistance of samples was tested three weeks after herbicide treatment through growth tests: two resistant biotypes (RES1 and RES2) and one susceptible biotype (SUS) were identified. Following an extensive bibliographic search, we selected twelve candidate genes reported in the literature as being involved in herbicide detoxification in *Echinochloa* spp., and analyzed their expression profiles before and after profoxydim treatment.

By means of an extensive bioinformatic analysis using the psRNATarget: A Plant Small RNA Target Analysis web server [42,43,44], on the basis of the nucleotide sequence of each candidate gene, we predicted in silico a set of miRNAs able to target their transcripts. Among these, none had already been identified in *Echinochloa* spp. miRNAs with a complementarity degree ≥ 80% with candidate gene transcripts were selected for analysis in our study. *Ata-miR166c-5p* was identified in *Aegilops tauschii* Coss., *ath-miR396b* and *ath-miR847* were identified in *Arabidopsis thaliana* (L.) Heynh, *gma-miR396f* was identified in *Glycine max* (L.) Merr., *gra-miR7486c* and *gra-miR8759* were identified in *Gossypium raimondii* Ulbr., and *osa-miR395f*, *osa-miR5538*, *osa-miR166a-5p*, *osa-miR166d-5p*, *osa-miR2099-5p*, and *osa-miR396a-5p* were identified in *O. sativa*. In Table 1, the miRNAs and the corresponding target genes analyzed in *E. oryzicola* are reported.

The expression profiles of miRNAs and their targets were evaluated in herbicide-susceptible (SUS) and -resistant (RES1 and RES2) *E. oryzicola* biotypes before (BT) and after (AT) profoxydim treatment.

### 2.1. Expression of CYP81A and miRNAs Targeting Their Transcripts

**Figure 1:**
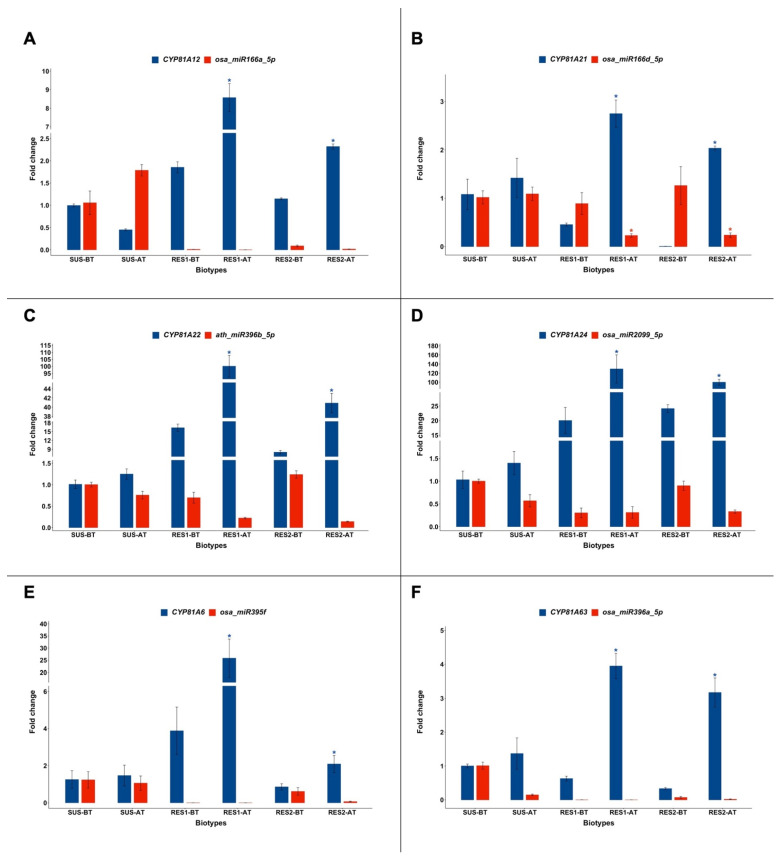
Expression levels of the miRNAs studied (red) and the mRNA of their target genes (blue) in susceptible (SUS) and resistant (RES1 and RES2) biotypes of *Echinochloa oryzicola* before (BT) and after (AT) profoxydim treatment. Mean and standard error values are indicated as bar plots and error bars, respectively. The expressions of genes and miRNAs were normalized with those of the respective internal references (*b-Actin* and *non-coding small nuclear RNA U6*). (**A**) *CYP81A12* and *osa-miR166a-5p*; (**B**) *CYP81A21* and *osa-miR166d-5p*; (**C**) *CYP81A22* and *ath-miR396b*-*5p*; (**D**) *CYP81A24* and *osa-miR2099-5p*; (**E**) *CYP81A6* and *osa-miR395f*; (**F**) *CYP81A63* and *osa-miR396a-5p*. *t*-test: *: *p* < 0.05.

Results concerning cytochromes P450 belonging to the 81A subfamily and miRNAs targeting their transcripts are reported in Figure 1. Significant changes in the expression levels (fold change > 2) of genes and miRNAs are identified with a *p* < 0.05.

As shown in Figure 1A, after profoxydim spraying, in the SUS biotype the expression of *CYP81A12* decreased by half (from 1.00 ± 0.05 to 0.45 ± 0.03), while that of *osa-miR166a-5p* increased (from 1.06 ± 0.46 to 1.79 ± 0.22). Meanwhile, gene expression significantly increased in RES1 (from 1.86 ± 0.21 to 8.58 ± 1.31) and RES2 (from 1.14 ± 0.03 to 2.32 ± 0.09), while *osa-miR166a-5p* transcription decreased.

As shown in Figure 1B, after herbicide application, *CYP81A21* expression remained almost equal in the SUS biotype, while it significantly increased in RES1 (0.45 ± 0.05 vs. 2.75 ± 0.48) and in RES2 (0.006 ± 0.001 vs. 2.00 ± 0.01). The expression of *osa-miR166d-5p*, which displayed similar values before treatment (~1.00) in all biotypes, remained almost equal in SUS, while significantly it decreased after profoxydim administration in RES1 and RES2.

As shown in Figure 1C, after herbicide spraying, the expression of *CYP81A22* mRNA slightly rose in the SUS biotype (1.01 ± 0.16 vs. 1.24 ± 0.20), while it significantly increased in RES1 (16.35 ± 2.17 vs. 100.10 ± 13.42) and in RES2 (7.97 ± 1.08 vs. 40.87 ± 3.69). *Ath-miR396b* transcription decreased in all biotypes after treatment.

A similar result was observed concerning *CYP81A24* and *osa-miR2099-5p* expression (Figure 1D). After herbicide application, gene expression slightly increased in the SUS biotype (1.03 ± 0.33 vs. 1.39 ± 0.44), while it significantly increased in both RES1 (20.04 ± 7.69 vs. 129.18 ± 53.58) and RES2 (24.12 ± 2.31 vs. 100.02 ± 11.19). *Osa-miR2099-5p* transcription was therefore found not to be triggered by profoxydim spraying in SUS, RES1, and RES2.

The expression of *CYP81A6* (Figure 1E) increased in SUS (1.25 ± 0.82 vs. 1.46 ± 0.97) and significantly rose in the RES1 (from 3.87 ± 2.22 to 25.80 ± 13.67) and RES2 (form 0.85 ± 0.29 to 2.09 ± 0.79) biotypes after herbicide spraying. Meanwhile, *osa-miR396a-5p* transcription displayed almost equal values in the SUS biotype before and after profoxydim administration, while it decreased in the RES1 and RES2 biotypes.

*CYP81A63* expression and *osa-miR396a-5p* transcription after herbicide treatment (Figure 1F) displayed profiles similar to those analyzed above: gene expression rose in SUS (1.00 ± 0.09 vs. 1.37 ± 0.79) and significantly increased in RES1 (0.63 ± 0.12 vs. 3.94 ± 0.64) and in RES2 (0.33 ± 0.05 vs. 3.17 ± 0.74), while *osa-miR396a-5p* transcription decreased.

### 2.2. Expression of CYP71A, CYP72A, EcGST, GSTF1, and eIF4B and of the miRNAs Targeting Their Transcripts

**Figure 2:**
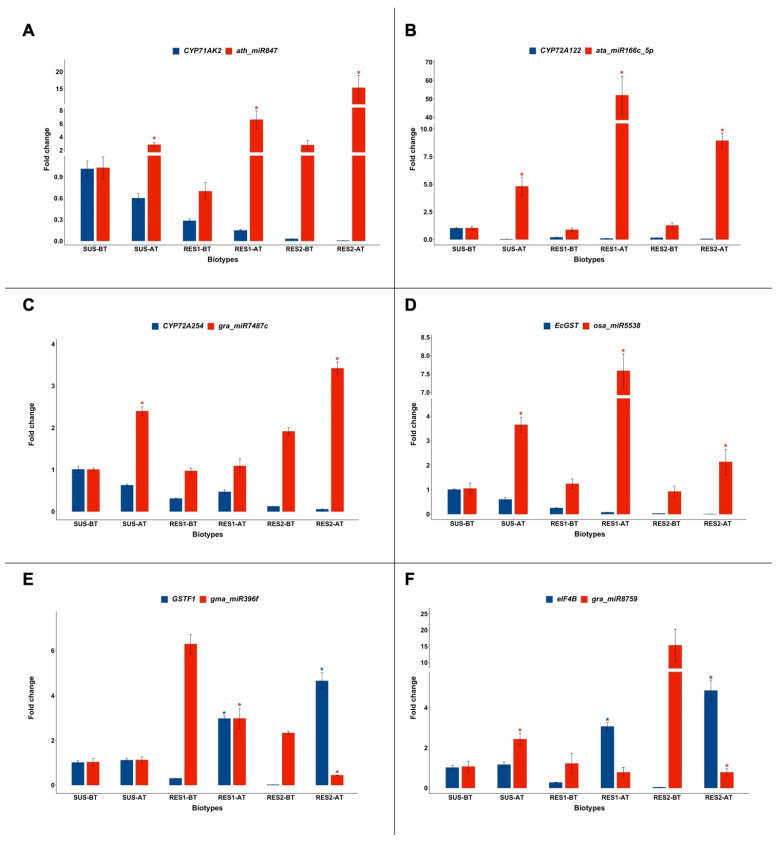
Expression levels of the miRNAs studied (red) and the mRNA of their target genes (blue) in susceptible (SUS) and resistant (RES1 and RES2) biotypes of *Echinochloa oryzicola* before (BT) and after (AT) profoxydim treatment. Mean and standard error values are indicated as bar plots and error bars, respectively. The expressions of genes and miRNAs were normalized with those of the respective internal references (*b-Actin* and *non-coding small nuclear RNA U6*). (**A**) *CYP71AK2* and *ath-miR847*; (**B**) *CYP72A122* and *ata-miR166c-5p*; (**C**) *CYP72A254* and *gra-miR7487c*; (**D**) *EcGST* and *osa-miR5538*; (**E**) *GSTF1* and *gma-miR396f*; (**F**) *eIF4B* and *gra-miR8759*. *t*-test: *: *p* < 0.05.

Results concerning cytochromes P450 belonging to the 71A and 72A subfamilies, *EcGST*, *GSTF1*, *eIF4B*, and the miRNAs targeting their transcripts are reported in Figure 2. Significant changes in the expression levels (fold change > 2) of genes and miRNAs are identified with a *p* < 0.05.

The expression of *CYP71AK2* (Figure 2A) showed very low levels (≤1.00) in the SUS, RES1, and RES2 biotypes before profoxydim application and further decreased after spraying. Meanwhile, after herbicide treatment, *ath-miR847* transcription significantly rose in the SUS (from 1.02 ± 0.26 to 2.82 ± 0.63), RES1 (from 0.69 ± 0.21 to 6.58 ± 2.30), and RES2 (from 2.76 ± 1.25 to 15.25 ± 6.32) biotypes.

Low values of *CYP72A122* expression (Figure 2B) were recorded in the SUS, RES1, and RES2 biotypes both before and after profoxydim administration (≤1.00), while *ata-miR166c-5p* transcription significantly rose (*p* < 0.05) in SUS (from 1.02 ± 0.26 to 4.79 ± 1.45), RES1 (from 0.87 ± 0.28 to 51.91 ± 17.90), and RES2 (from 1.25 ± 0.39 to 8.93 ± 1.18) after herbicide application.

Similar expression profiles were shown for *CYP72A254* and *gra-miR7486c* (Figure 2C), with the *CYP72A254* mRNA being under-expressed in the SUS, RES1, and RES2 biotypes after herbicide application (≤1.00 fold) and *gra-miR7486c* being overexpressed in SUS (1.00 ± 0.06 vs. 2.39 ± 0.17; *p* < 0.05), RES1 (0.96 ± 0.12 vs. 1.08 ± 0.30; *p* > 0.05), and RES2 (1.91 ± 0.15 vs. 3.41 ± 0.26; *p* < 0.05) after herbicide application.

*EcGST* (Figure 2D) showed low expression values in all of the analyzed biotypes, both before and after profoxydim treatment (≤1.00), with its expression further decreasing after spraying. *Osa-miR5538* expression displayed appreciable values even before treatment, and significantly increased (*p* < 0.05) after herbicide administration in SUS (from 1.04 ± 0.38 to 3.64 ± 0.53), RES1 (from 1.23 ± 0.34 to 7.58 ± 0.80), and RES2 (from 0.91 ± 0.39 to 2.12 ± 0.90).

In the SUS biotype, both *GSTF1* mRNA and *gma-miR396f* expression (Figure 2E) were found to be unaffected by herbicide treatment, while in resistant biotypes, the expression of *GSTF1* mRNA significantly increased (*p* < 0.05) in RES1 (from 0.29 ± 0.006 to 2.97 ± 0.19) and in RES2 (from 0.01 ± 0.001 to 4.65 ± 0.62) after profoxydim application. Meanwhile, *gma-miR396f* expression significantly decreased after herbicide spraying (6.28 ± 0.73 vs. 2.97 ± 0.79 for RES1 and 2.32 ± 0.13 vs. 0.43 ± 0.01 for RES2).

In Figure 2F, the expression profiles of *eIF4B* and *gra-miR8759* are reported. It can be observed that, in the SUS biotype, the expression of *eIF4B* did not vary after treatment, while it significantly increased in RES1 (0.26 ± 0.03 vs. 3.06 ± 0.34) and RES2 (0.03 ± 0.003 vs. 4.85 ± 0.90). Profoxydim application induced a significantly increased expression of *gra-miR8759* in the SUS biotype, non-significantly decreased transcription in RES1 (from 1.21 ± 0.89 to 0.77 ± 0.44), and significantly decreased transcription in RES2 (from 15.29 ± 8.60 to 0.77 ± 0.31).

## 3. Discussion

In the present study, we analyzed the microRNA-mediated regulation of enzymes involved in herbicide detoxification in *Echinochloa oryzicola* (Vasinger) Vasinger (late-watergrass). The aim was to understand some manifestations of herbicide resistance not related to the commonly known mechanisms (i.e., TSR). In particular, we wanted to explore the role of sncRNAs in this phenomenon.

For this purpose, the expression of cytochromes P450 monooxygenase, glutathione S-transferase, and eukaryotic translation initiation factor 4B and that of miRNAs able to target mRNA of the same genes was analyzed before and after profoxydim treatment in susceptible (SUS) and resistant (RES1 and RES2) biotypes. Our results highlight that some of the miRNAs we identified can affect profoxydim resistance in *E. oryzicola* collected from rice fields in the Lombardy region (Po Valley, northern Italy). Herbicide application triggered the transcription of some miRNAs which down-regulated the expression of target genes, reducing their detoxification ability. Otherwise, when herbicide spraying did not stimulate the transcription of miRNAs, the target gene mRNAs could be translated into proteins, leading to herbicide detoxification.

It is known that epigenetics participates in stress responses. For example, chromatin modifications have been described when plants are exposed to stressful environments [28]. The role of sncRNAs in the adaptation of plants to chemical control (i.e., herbicide application) has only been considered recently. For example, Pan et al. (2016) analyzed how miRNAs regulated the expression of genes involved in resistance to fenoxaprop-P-ethyl in *Beckmannia syzigachne* (Steud.) Fernald [45]. Żywicki et al. (2015) recorded a significantly altered expression of several miRNA families upon treatment of maize (*Z. mays*) with glyphosate [46]. Cusaro et al. (2022) investigated in *E. crus-galli* the ability of some miRNAs to affect the expression of CYP450, GST, and *eIF4B*, all involved in bispyribac-Na (ALS inhibitor) detoxification [36]. In light of these first observations, in the present study, we wanted to search for an epigenetic miRNA-mediated response to profoxydim (ACCase inhibitor) treatment in *E. oryzicola*.

**Figure 3:**
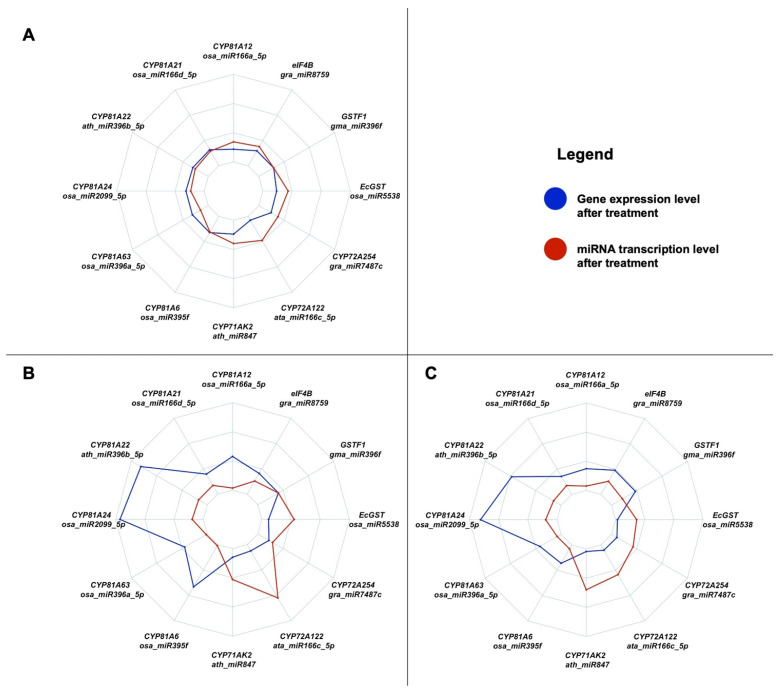
Radar chart of the expression profiles of genes (blue line) and miRNAs (red line) in the SUS biotype (**A**), in the RES1 biotype (**B**), and in the RES2 biotype (**C**) after profoxydim treatment. ata—*Aegilops tauschii* Coss.; ath—*Arabidopsis thaliana* (L.) Heynh; gma—*Glycine max* (L.) Merr.; gra—*Gossypium raimondii* Ulbr.; osa—*Oryza sativa* L.

Figure 3 graphically summarizes the results regarding the expression of the studied miRNAs and of their target genes after the application of profoxydim in susceptible (SUS) and resistant (RES1 and RES2) biotypes.

The expression of *ata-miR166c-5p*, *ath-miR847*, *osa-miR5538*, *gra-miR7487c*, *osa-miR166a-5c*, and *gra-miR8759* was stimulated in the SUS biotype after herbicide application. On the other hand, reduced expression of their respective target genes (*CYP72A122*, *CYP71AK2*, *EcGST*, *CYP72A254*, *CYP81A12*, and *eIF4B*) was observed (Figure 3A). Considering the RES biotypes, the expression of *ata-miR166c-5p*, *ath-miR847*, *osa-miR5538*, and *gra-miR7487c* was highly stimulated after profoxydim administration, and the expression of their target genes (*CYP72A122*, *CYP71AK2*, *EcGST*, *CYP72A254*) was reduced (Figure 3B,C). The expression of the remaining miRNAs (*osa-miR2099-5p*, *ath-miR396b*, *osa-miR395f*, *osa-miR396a-5p*, *osa-miR166a-5p*, *osa-miR166d-5p*, *gra-miR8759*, and *gma-miR396f*) was not stimulated by the herbicide, and the expression of their target genes (*CYP81A24*, *CYP81A22*, *CYP81A6*, *CYP81A63*, *CYP81A12*, *CYP81A21*, *eIF4B*, and *GSTF1*) did not appear to reduce after herbicide application. Hence, after profoxydim treatment, the expression of some genes (CYP450, 81A subfamily) was higher in the RES biotypes (Figure 3B,C) than in the susceptible one (Figure 3A).

Our findings are in agreement with Iwakami et al., 2014 [18] and with Iwakami et al., 2019 [17], who demonstrated the involvement of CYP450 of the 81A subfamily in the detoxification of ALS and ACCase inhibitors. In fact, it was reported that the overexpression of *CYP81A12* and *CYP81A21* confers cross-resistance to ALS (bensulfuron-methyl and penoxsulam) and ACCase (diclofop-methyl, tralkoxydim, pinoxaden) in *E. phyllopogon*, while *CYP81A24* is involved in the detoxification of cyclohexanedione (ACCase inhibitor), such as profoxydim. Furthermore, Dimaano et al. (2020) highlighted that in *E. phyllopogon, CYP81A12*, *CYP81A21*, and *CYP81A24’s* aminoacidic sequences are similar [13]. This fact would explain the synergistic action of these three proteins towards profoxydim. Iwakami et al. (2019) also reported how *CYP81A63* was involved in the metabolization of ACCase inhibitors in barley, cyclohexanedione included [17]. In our experimental conditions, we assessed that the transcription of some miRNAs (*osa-miR166a-5p*, *osa-miR166d-5p*, *osa-miR2099-5p*, and *osa-miR396a-5p*), selected for their ability to target the abovementioned genes, was not triggered by profoxydim, allowing the overexpression of cytochromes P450.

Iwakami et al. (2014) observed the overexpression (i.e., higher metabolizing activity) of *CYP71AK2*, *CYP72A122*, and *CYP72A254* in *E. phyllopogon* after treatment with bispyribac-Na (ALS inhibitor) [21]. In our present study, we found that *gma-miR396f*, *ata-miR166c-5p*, and *gra-miR7486c* targeting these cytochromes P450 were triggered following profoxydim application, causing a down-regulation of gene expression.

Overexpression of *GSTF1* and *eIF4B* and reduced transcription of *gra-miR8759* and *osa-miR5538* were observed after herbicide treatment. Our findings are in agreement with those of Dalazen et al. (2018) [23] and Li G. et al. (2013) [20], who reported an increased expression of the same genes in *E. crus-galli* resistant to imazethapyr (ALS inhibitor), as well as those of Cummins et al. (1999, 2003, 2013) [47,48,49], who observed an increased expression of GST genes in *Alopecurus myosuroides* Huds. and *Lolium rigidum* Gaudin resistant to chlorotoluron (inhibitor of photosynthesis at photosystem II) and fenoxaprop-P-ethyl (ACCase inhibitor). After treatment with profoxydim, we observed a reduced expression of *EcGST*, with an increased expression of *osa-miR395f* in the RES biotypes, suggesting the ability of this miRNA to down-regulate glutathione S-tranferase.

In our study, we considered target genes that are known to be involved in resistance in *Echinochloa* and are widely studied. Next, we searched in silico for miRNA sequences with a high matching rate with the target genes. The predicted miRNAs were finally validated in *E. oryzicola* by means of RT-qPCR. Moreover, we highlighted their role in the down-regulation of some genes involved in profoxydim resistance.

All of the miRNAs tested in this research and previously reported in other plant species [50,51,52,53,54,55,56,57] were also expressed in *E. oryzicola*. Moreover, the increased expression of *ata-miR166c-5p*, *ath-miR847*, *osa-miR5538*, and *gra-miR7487c*, along with the reduced expression of *CYP72A122*, *CYP71AK2*, *EcGST*, and *CYP72A254*, suggests the occurrence of a miRNA-mediated down-regulation of these detoxifying enzymes triggered by profoxydim.

Since it is known that the transcription of sncRNAs is influenced by ecological factors [27,28,29,30], our present findings provide the basis for future insights into the relationship between ecological factors and resistance occurrence. Planning new and sustainable control strategies aimed at reducing pesticide inputs also requires the assessment of the entire miRNAome of *E. oryzicola* in order to better understand the epigenetic regulation of proteins involved in herbicide resistance.

## 4. Materials and Methods

### 4.1. Plant Materials, Growth Conditions and Herbicide Treatment

Plant material collection was carried out in three rice fields in the Lombardy region (Po Valley, northern Italy). In two different experimental plots, seeds of *E. oryzicola* that survived profoxydim (AURA^®^—BASF SE, Ludwigshafen, Germany) treatment were collected. Seeds were also collected from plants in an untreated plot and were used as the susceptible reference.

In order to assess the actual resistance/susceptibility to profoxydim of the collected specimens, controlled growth trials were carried out. Seeds from the three rice fields were planted in a universal organic compound (Vigorplant Italia Srl, Fombio, Italy) in separate 100 mL pots. Germinated plants were maintained in a growth chamber with a mean temperature of 20 °C, relative humidity of 70%, and a photoperiod of 14/10 h (day/night). Three biological replicates were used for each treatment. At the three-leaf stage, profoxydim (AURA^®^) was sprayed on plants at the label dose of 0.4 L/ha with the addition of adjuvant (DASH HC^®^—BASF SE, Ludwigshafen, Germany) following the manufacturer’s instructions. A Honda WJR 2525 ET^®^ backpack sprayer (Honda Motor Co., Ltd., Minato, Tokyo, Japan) with a spray pressure of 4 bar and a flow rate of 43 mL/s, resulting in a spray volume of 300 L/ha, was used to apply the herbicide to the entire plant collection. Three weeks after treatment, the sensitivity of plants to profoxydim was tested both through visual inspection and through the determination of the weight of the biomass of collar-cut plants, following European and Mediterranean Plant Protection Organization (EPPO) standards [58,59]. Growth tests thus made it possible to identify the susceptible (SUS) and resistant biotypes (RES1 and RES2).

For each biotype, leaves were collected from 3 plants in each pot, just before (BT) and 24 h after treatment (AT), and stored at −40 °C until DNA and RNA extraction.

### 4.2. DNA Extraction and Assessment of ACCase Gene Mutations

Genomic DNA was extracted from the frozen leaf tissues of each biotype, using the DNeasy Plant Kit (QIAGEN SpA, Hilden, Germany) according to the manufacturer’s instructions. DNA quality and concentration were assessed by means of electrophoresis on 2% agarose gel stained with ethidium-bromide and using a fluorometric method (Qubit fluorometer, Life Technologies, Carlsbad, CA, USA) according to the manufacturer’s protocol. In samples with a low DNA quantity, extraction was repeated. On average, extracted DNA concentration was around 80 ng/µL. DNA was stored at −20 °C.

Restriction fragment length polymorphism (RFLP) analysis was used to detect mutations at the Ile-1781 codon and at the Ile-2041 codon of the *Echinochloa* spp. ACCase gene involved in TSR. The ACCase gene (GeneBank a.n.: HQ395759.1) was PCR-amplified using universal primers (ACCase—Forward: 5′-CAGCYTGATTCCCAYGAGCGYT-3′; ACCase—Reverse: 5′-CCATGCAYTCTTYGAGYTCCTCTGA-3′) by Déyle et al., 2005 [60]. The reaction was carried out in a T100 thermal cycler (BIO-RAD, Hercules, CA, USA) in a 10 μL volume containing 2 μL of genomic DNA (20 ng), 2.4 μL (0.8 U) of GoTaq^®^ Hot Start Green Master Mix (Promega, Madison, WI, USA), 0.6 μL of each primer (1 μM), 0.5 μL of MgCl_2_ (2 mM), and 2 μL of nuclease-free H_2_O. The PCR profile set was as follows: an initial denaturation step (5 min at 95 °C) followed by 35 cycles (45 s at 95 °C, 45 s at 57 °C, and 45 s at 72 °C) and a final extension step (10 min at 72 °C). The amplification products were digested with XapI (Thermo Fisher Scientific, Waltham, MA, USA) endonuclease to detect mutation at the Ile-1781 codon or with EcoRI endonuclease (Promega) to detect mutation at the Ile-2041 codon. Digestion was performed at 37 °C for 24 h in a total volume of 15 μL containing 3 μL of the PCR product (200 ng), 1 μL of TAE buffer, 1 μL (12 U) of restriction enzyme, and 10 μL of nuclease-free H_2_O. The resulting products were resolved on 2% agarose gel (Certified Molecular Biology Agarose—BIO-RAD) stained with ethidium bromide. Amplicon size was determined using a 100 bp DNA ladder (Promega) using the GelDoc Go Gel Imaging System (BIO-RAD).

Selective amplification and digestion of the ACCase gene allowed us to identify wild-type late-watergrass plants for the following gene and miRNA expression analysis.

### 4.3. RNA Extraction and cDNA Synthesis

Both before (BT) and after (AT) profoxydim spraying, from the susceptible (SUS) and resistant (RES1 and RES2) biotypes, total RNA was extracted from a pool of frozen leaf tissues obtained from 3 different plants (100 mg each) per pot. The RNeasy Plant Kit (QIAGEN SpA) was used, following the manufacturer’s instructions. RNA concentration and quality were measured using the Qubit RNA Assay Kit on a Qubit 3.0 Fluorometer (Life Technologies) according to the manufacturer’s protocol. In samples where RNA concentration was below the instrument detection threshold, extraction was repeated. On average, the extracted RNA concentration was around 100 ng/µL. RNA was conserved at −80 °C.

cDNA was obtained following a reverse transcription reaction with the miRCURY LNA RT Kit (QIAGEN SpA). The reaction mixture contained 10 μL of RNA template (5 ng/µL), 4 μL of 5× miRCURY RT reaction buffer, 2 µL of 10× miRCURY RT enzyme mix, and 10 µL of nuclease-free H_2_O. The reverse transcription reaction was performed at 37 °C for 60 min, followed by 95 °C for 10 min. cDNA was stored at −20 °C.

### 4.4. Analyzed Genes and miRNA Prediction and Validation

Genes involved in herbicide detoxification were selected on the basis of previously published studies [16,17,18,21,23,61]. We analyzed the expression of nine cytochromes P450 monooxygenase (*CYP81A12*, *CYP81A21*, *CYP81A22*, *CYP81A24*, *CYP81A63*, *CYP81A6*, *CYP71AK2*, *CYP72A122*, and *CYP72A254*), two glutathione S-transferase (*EcGST* and *GSTF1*), and the eukaryotic translation initiation factor 4B (*eIF4B*). The sequences of the most conserved DNA regions of each gene were obtained in Gene Bank [62] and in GrainGenes—A Database for Triticeae and Avena [63]. The primer sequences of the selected genes were designed using Primer-BLAST [64]. The primer selection was based on default parameters, with the exception of annealing temperature, which was set at 60 °C; the primer length was set around 20 bp, and the expected PCR product size was set from 70 bp to 150 bp.

Table 2 lists the primers used to analyze the expression of CYP450, GST, and *eIF4B* genes in *E. oryzicola*.

According to the methodology of Suddal et al., 2024 [65], miRNA prediction was carried out in silico using the psRNATarget: A Plant Small RNA Target Analysis web server (Schema V2, 2017 release) [42,43,44]. Starting from the complete coding sequence of genes, in “.fasta” format (Table 2), optimal alignment between miRNAs and target genes was assessed by applying the Smith–Waterman algorithm [66] using the default parameters. Mature miRNAs with a sequence complementarity rate ≥ 80% with the target gene and annotated in miRbase [67] for other plants were selected (Table 3). According to Kulcheski et al., 2011 [68], the predicted miRNAs were finally validated in *E. oryzicola* via RT-qPCR.

### 4.5. RT-qPCR Analysis

The expression profiles of the genes and miRNAs considered were determined using relative real-time quantitative PCR (RT-qPCR) The amplification was carried out using the SYBR Green^®^ kit (Takara Holdings Inc., Shimogyōku, Kyoto, Japan) with the Applied Biosystems 7300 Real-Time PCR System (Thermo Fisher Scientific, Waltham, MA, USA) on 96-well plates (PCR-96M2-HS-C^®^, Axygen Scientific—part of Thermo Fisher Scientific, Waltham, MA, USA).

For the analysis of genes, RT-qPCR was performed in a total volume of 10 μL containing 2 μL of DNA (5 ng/µL) and 8 μL of master mix composed of 5 μL of TB Green Prmix Ex Taq (Tli RNaseH Plus, Takara Holdings Inc.), 0.5 μL (1 μM) of forward and reverse primers, 0.5 μL of ROX Reference Dye, and 1.5 µL of nuclease-free H_2_O for each sample. The amplification program comprised an initial incubation at 95 °C for 30 s, followed by 40 cycles of amplification (95 °C for 5 s, 60 °C for 30 s). A dissociation cycle was carried out at 95 °C for 15 s, 60 °C for 1 min, and 95 °C for 15 s, followed by increasing the temperature stepwise by 0.3 °C.

For the analysis of miRNAs, RT-qPCR was carried out in a total volume of 10 μL containing 2 μL of cDNA (1 ng/µL) and 8 μL of master mix composed of 5 μL of TB Green Prmix Ex Taq (Tli RNaseH Plus) (Takara Holdings Inc.), 1 µL of miRCURY LNA miRNA (QIAGEN SpA), 0.5 μL of ROX Reference Dye, and 1.5 µL of nuclease-free H_2_O for each sample. The amplification program comprised an initial incubation at 95 °C for 30 s, followed by 40 amplification cycles (95 °C for 5 s, 60 °C for 31 s). A dissociation cycle was carried out at 95 °C for 15 s, 60 °C for 1 min, and 95 °C for 15 s, followed by increasing the temperature stepwise by 0.3 °C.

The threshold values (Ct) were determined by means of the 7300 Real-Time PCR System’s on-board software. Each sample was tested in triplicate.

### 4.6. Statistical Analysis

The comparative Ct method (2^−ΔΔCt^ method) by Livak and Schmittgen (2001) was used to calculate the expression levels of genes and miRNAs [69]. The relative expression of mRNAs was assessed using the *b-Actin* housekeeping gene (GeneBank a.n.: HQ395760.1) as an internal gene reference. The relative expression of miRNAs was assessed using *non-coding small nuclear RNA U6* (GeneBank a.n.: AT3G14735.1–NR141593.1) as an internal reference. The references used are considered among the best ones for relative quantitative expression according to different studies in plants [70,71,72,73].

The relative expression was calculated according to the ΔΔCt method using the following equation:

ΔΔCt = (Ct _target_ − Ct _reference_) − (Ct _calibrator_ − Ct _reference_), (1)

where susceptible biotypes before treatment (SUS-BT) were considered as a calibrator [69].

The expression levels of genes and miRNAs, calculated in triplicate for susceptible (SUS) and resistant (RES1 and RES2) late-watergrass biotypes, were analyzed as means and standard errors calculated from three replicates. The expressions of genes and miRNAs were normalized with those of the respective internal references [69].

The relative expression values (fold change) and standard errors of candidate genes and miRNAs were graphed as bar plots using the function “ggbarplot” of the package ggpubr [74] in the R 4.3.0 software [75].

Significant differences in expression levels of candidate metabolic genes and relative miRNAs before and after treatment were analyzed using the *t*-test in R 4.3.0 software [75].

Radar charts were graphed using the function “radarchart” of the package “fmsb” [76] in R 4.3.0 software [75].

## 5. Conclusions

This research is the first carried out on Italian *E. oryzicola* focusing on the miRNA-mediated epigenetic regulation of genes involved in profoxydim resistance.

Our findings highlight that herbicide treatment can trigger miRNA transcription, causing a down-regulation in the expression of target genes (i.e., *ata-miR166c-5p* vs. *CYP72A122*, *ath-miR847* vs. *CYP71AK2*, *osa-miR5538* vs. *EcGST*, and *gra-miR7487c* vs. *CYP72A254*). These results are a basis for further investigations of epigenetic regulation mediated by miRNAs as part of studies aimed at obtaining more information on the mechanisms of herbicide resistance.

At present, the entire miRNAome of *E. oryzicola* is not available. The availability of a large set of miRNAs able to affect herbicide detoxification would be useful to provide deeper insights into the complex mechanisms underlying the resistance phenomenon.

The methodological analysis of our present study represents an innovative approach to assessing epigenetic mechanisms affecting herbicide resistance and could be applied to other crops, such as in vineyards, where, in recent years, the evolution of weeds resistant to the most commonly used herbicides (i.e., glyphosate) has been observed.

A better understanding of the epigenetic regulation of herbicide resistance will be useful for planning more targeted and sustainable methods for controlling this phytosanitary threat, reducing inputs, and optimizing precision weed management (PWM) technologies. Moreover, it can be hypothesized that miRNAs could be applied as alternative biopesticides in modern agriculture due to their ability to migrate among organisms and regulate specific genetic processes.

Our results highlighted the miRNA-mediated post-transcriptional regulation of the expression of genes responsible for resistance. Since miRNAs are known to be influenced by ecological factors, conducting tests under controlled conditions to evaluate which biotic and abiotic factors (i.e., climate change, edaphic properties, soil microbial communities) may play a role in influencing miRNA-mediated resistance would be a favorable strategy to obtain new information that is useful for predicting the occurrence of resistance, favoring a proactive approach to handle this phytosanitary threat.

## Figures and Tables

**Table 1:** miRNAs and the corresponding target genes analyzed in *Echinochloa oryzicola*. In silico miRNA prediction was carried out using the psRNATarget: A Plant Small RNA Target Analysis web server. Optimal alignment between miRNAs and the target gene was investigated by applying the Smith–Waterman algorithm, using the default parameters of the server (Schema V2, 2017 release). Only miRNAs with a high sequence complementarity rate (≥80%) with the target gene were considered for the study.

miRNA	Target Gene
*osa-miR166a-5p*	*CYP81A12*
*osa-miR166d-5p*	*CYP81A21*
*ath-miR396b*	*CYP81A22*
*osa-miR2099-5p*	*CYP81A24*
*osa-miR396a-5p*	*CYP81A63*
*ath-miR847*	*CYP81A6*
*gma-miR396f*	*CYP71AK2*
*ata-miR166c-5p*	*CYP72A122*
*gra-miR7486c*	*CYP72A254*
*gra-miR8759*	*GSTF1*
*osa-miR395f*	*EcGST*
*osa-miR5538*	*eIF4B1*

ata—*Aegilops tauschii* Coss.; ath—*Arabidopsis thaliana* (L.) Heynh.; gma—*Glycine max* (L.) Merr.; gra—*Gossypium raimondii* Ulbr.; osa—*Oryza sativa* L.

**Table 2:** Nucleotide sequences of the primers used for RT-qPCR analysis of CYP450, GST, and *eIF4B* gene expression in *Echinochloa oryzicola*.

Gene ID	Primer Sequence (5′-3′)	Reference
*CYP81A12*	F: tgagctcttccatcgtcgtg R: tactttttggcgactccgct	Iwakami et al., 2014 [18]
*CYP81A21*	F: tagcatcatccacgagacgc R: tacacgttcaccagcagcat	Iwakami et al., 2014 [18]
*CYP81A22*	F: cggcgcgctggtccagtt R: tgacatgagcagttccatcg	Iwakami et al., 2014 [18]
*CYP81A24*	F: gaggtctacaccgatgccac R: cttcttgagcttctccgggt	Iwakami et al., 2014 [18]
*CYP81A63*	F: gagaccatcgctcagaccaa R: atcttgttcctcacgccgaa	Dimaano et al., 2020 [13] Iwakami et al., 2019 [17]
*CYP81A6*	F: gactattcaacccgggcgat R: caagttctgcacggcaagag	Pan et al., 2022 [61]
*CYP71AK2*	F: acgtgtgggacaagttcctg R: ggctttgatgcgatcgtctg	Iwakami et al., 2014 [21]
*CYP72A122*	F: agttcaagccggagaggttc R: catcttggcttcaagcagcg	Iwakami et al., 2014 [21]
*CYP72A254*	F: ttacgaggtactccggctgt R: gtcagggtcgtggtgaatgt	Iwakami et al., 2014 [21]
*EcGST*	F: gccgaggaggacctgaagaac R: gtgactcacagataggcttaccgt	Li et al., 2013 [16]
*GSTF1*	F: tgcctcttcaaccccatgat R: aggtactcgtgctgggagag	Dalazen et al., 2018 [23]
*eIF4B1*	F: cgagcagcttacaagggact R: gtggttccataccaccacga	Dalazen et al., 2018 [23]

**Table 3:** miRNAs selected for expression analysis in *Echinochloa oryzicola*.

Name	a.n.	miRNA Sequence (5′-3′)	Reference
*ata-miR166c-5p*	MIMAT0037248	ggaacguuggcuggcucgagg	Jia et al., 2013 [56]
*ath-miR396b*	MIMAT0000945	uuccacagcuuucuugaacuu	John-Rohades et al., 2004 [50]
*ath-miR847*	MIMAT0004278	ucacuccucuucuucuugaug	Rajagopalan et al., 2006 [51]
*gma-miR396f*	MIMAT0021069	agcuuucuugaacuucuuaugccua	Radwan et al., 2011 [54]
*gra-miR7486c*	MIMAT0034235	uuuguccacgugaacagaaaacgc	Xue et al., 2013 [57]
*gra-miR8759*	MIMAT0034189	ugguggaaguauugugcccgg	Xue et al., 2013 [57]
*osa-miR395f*	MIMAT0000974	gugaauuguuugggggaacuc	John-Rohades et al., 2004 [50]
*osa-miR5538*	MIMAT0022174	acugaacucaaucacuugcugc	Wei et al., 2011 [55]
*osa-miR166a-5p*	MIMAT0022855	ggaauguugucugguucaagg	Du et al., 2011 [53]
*osa-miR166d-5p*	MIMAT0022858	ggaauguugucuggcucgagg	Du et al., 2011 [53]
*osa-miR2099-5p*	MIMAT0010062	ugaauauguuuguacaagcuuu	Xue et al., 2009 [52]
*osa-miR396a-5p*	MIMAT0000977	uuccacagcuuucuugaacug	Du et al., 2011 [53]

ata—*Aegilops tauschii* Coss.; ath—*Arabidopsis thaliana* (L.) Heynh.; gma—*Glycine max* (L.) Merr.; gra—*Gossypium raimondii* Ulbr.; osa—*Oryza sativa* L.; a.n.: miRbase accession number.

## Data Availability

The raw data supporting the conclusions of this article will be made available by the authors, without undue reservation.
